# Case of Osseous Deposit within the Nervous Pulp of a Molar Tooth

**Published:** 1850-11

**Authors:** S. S. Hornor

**Affiliations:** Dentist; Philadelphia


### *Case of Osseous Deposit within the nervous pulp of a Molar Tooth.*

By S. S. Hornor, Dentist.

About two months since, I was waited on by a young lady, a member of one of our most respectable families for the purpose of having the first superior molar tooth (left side) filled. On examination, the tooth presented but a slight decay, yet it was so exceedingly sensitive, as to require a mild application for the purpose of allaying the sensibility before filling it; after which I succeeded in plugging it with gold, to my satisfaction, and as I had reason to hope, effectually preserved the tooth.

On Monday last, however, I was called to see her, when she complained of constant pain in the tooth, and was also suffering from a bilious attack, for which my eminent friend, Prof. Mitchell, was attending her.

As she was unwilling to submit to leeching, an opium plaster was prescribed, without the desired effect, and on Wednesday last,

I extracted the tooth, which I found highly inflamed, the nerve entirely dead, and the periosteum of the fangs in a suppurative state. Upon further inspection, its singular appearance induced me to break it, for the purpose of examining the nervous pulp, which had assumed the character of a gristly mass, of a blood-red color, surrounded by a sero-sanguinolent liquid, containing in the very centre, and constituting about two-thirds of the whole mass, a semi-transparent bony substance, so hard as to resist the point of a penknife.

After freeing the bone from the surrounding substance, and placing it under the field of a microscope, of moderate power, it presented the appearance of a transparent and irregular pebble, with many projecting points, beautifully rounded off.

Oudet describes bony formations within the tooth from altered secretions of the pulp, in Dictionnaire de Médecine, vol. 1, p. 186, but, this is the first case of the kind ever met with in my own practice. I have therefore, taken the liberty of sending you a description of it, with a request that you will give it a place in your valuable journal.

*Philadelphia, Sep. 21st,* 1850.

